# Enhancing productivity of Chinese hamster ovary (CHO) cells: synergistic strategies combining low-temperature culture and mTORC1 signaling engineering

**DOI:** 10.3389/fbioe.2023.1268048

**Published:** 2023-11-21

**Authors:** Farzaneh Shahabi, Shahriyar Abdoli, Zahra Bazi, Fatemeh Shamsabadi, Ahad Yamchi, Majid Shahbazi

**Affiliations:** ^1^ Medical Cellular and Molecular Research Center, Golestan University of Medical Sciences, Gorgan, Iran; ^2^ Department of Medical Biotechnology, School of Advanced Technologies in Medicine, Golestan University of Medical Sciences, Gorgan, Iran; ^3^ Department of Plant Breeding and Biotechnology, College of Plant Production, Gorgan University of Agriculture Science and Natural Recourses, Gorgan, Iran; ^4^ AryaTina Gene (ATG) Biopharmaceutical Company, Gorgan, Iran

**Keywords:** bioprocess engineering, mTORC1, CHO cells, thermal shift, HSP90 promoter

## Abstract

**Introduction:** The growing demand for recombinant proteins in medicine has prompted biopharmaceutical companies to seek ways to maximize the manufacturing process. Despite its known negative impact on cell growth, temperature shift (TS) has emerged as a cost-effective strategy to enhance protein quantity and quality in Chinese Hamster Ovary cells (CHO). As cells adapt their growth and protein synthesis rate to the environment through influencing mTOR complex 1 (mTORC1), here we evaluated the potential of mTORC1 signaling engineering to improve the production of granulocyte-macrophage colony-stimulating factor (GM-CSF) protein in stable CHO cells at low temperature.

**Methods:** First, the expression of genes that negatively control mTORC1 functions in response to environmental fluctuations, including TSC1, AMPK, MAPKAPK5, and MARK4 genes, was assessed via real-time qPCR in CHO-K1 after a temperature shift from 37°C to 30°C. Then, plasmids harboring the shRNAs targeting these genes were constructed into the PB513B-1 plasmid with expression driven by either the constitutive CMV promoter or the cold-inducible HSP90 promoter. Finally, the impact of transient gene downregulation was evaluated on GM-CSF and mTOR proteins productivity in GM-CSF-producing CHO-K1 cells using ELISA and Western-blot assays, respectively. The growth rate of the transfected cells at the two temperatures was evaluated using flow cytometry.

**Results:** Hypothermic conditions promote the upregulation of mTORC1 inhibitor genes, especially TSC1 and MAPKAPK5, while downregulating S6K, a key effector of the mTORC1 signaling pathway, in CHO-K1 cells. Transcription and protein levels of mTOR increased upon transfection, “pB513-b CMV-P/4shRNAs/GFP” plasmid, “pB513-bHSP90-P/4sh-RNAs/GFP” and pB513B-1 plasmid as mock group in GM-CSF-producing CHO-K1 cells (approximately 60%), along with a high transcript level of S6K. Cell growth-related characteristics were improved, albeit with distinct effects at different temperatures. Notably, these changes were more efficient at 30°C when utilizing the HSP90 promoter, resulting in a three-fold increase in GM-CSF production after 3 days.

**Conclusion:** This study highlights the importance of temperature regulation and mTORC1 modulation in CHO cellular processes, particularly in recombinant protein production. Understanding these mechanisms paves the way for developing innovative strategies to enhance cell growth, protein synthesis, and overall bioprocess performance, particularly in manufacturing human therapeutic proteins.

## Graphical Abstract

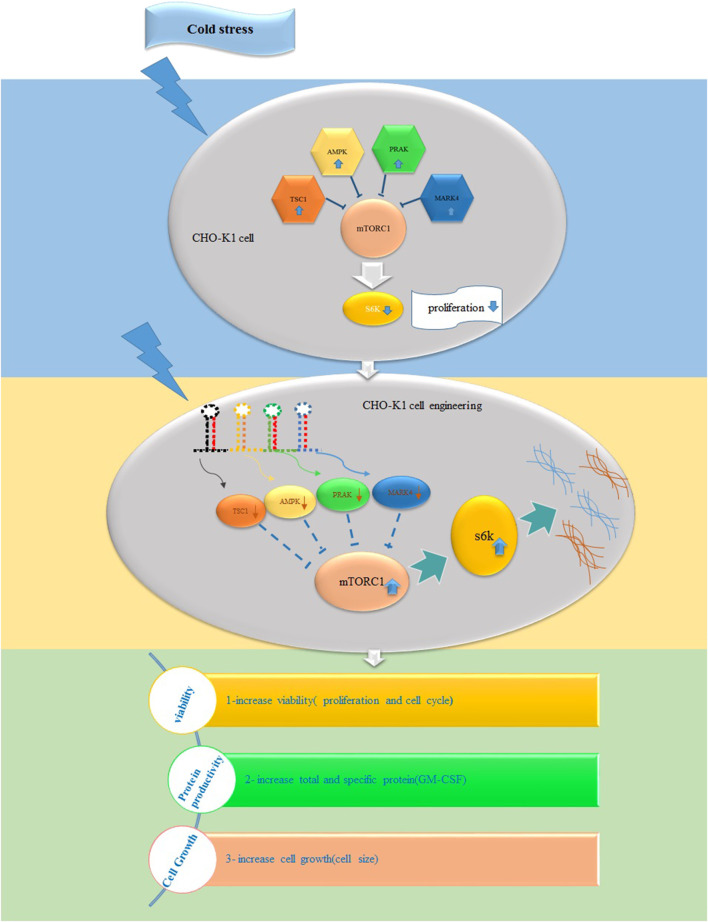

## 1 Introduction

Chinese hamster ovary (CHO) cells are the most commonly used mammalian expression system for manufacturing protein-based biopharmaceutical products. Compared to other mammalian expression systems, they offer distinct advantages, including safety, high specific productivity (qp), effective post-translational modifications, ease of cultivation in large bioreactor and serum-free media, and flexibility for genetic modification (Sellick et al., 2011; Bryan et al., 2021). However, the cost of producing recombinant proteins in CHO cells remains relatively high compared to microbial host systems. This cost disparity is primarily due to their sensitivity to environmental factors influencing their productivity, growth, and viability in bioreactors (Bryan et al., 2021; Yao et al., 2021). In recent times, culturing CHO cells at low temperatures provides the considerable potential for enhancing recombinant protein production in the industry (Al-Fageeh et al., 2006; McHugh et al., 2020; Nguyen et al., 2020). Temperature shift (TS) can boost cell-specific productivity by enhancing the stability of mRNAs and transcription levels and improving protein folding. Additionally, it reduces total cell metabolism, leading to a decline in the production of toxic substances, modulation of the effects of nutrition deprivation, and increased cell viability (Yee et al., 2009; Kantardjieff et al., 2010; Lin et al., 2015).

Furthermore, the development of endogenous inducible promoters utilizing cold-induced promoters has garnered attention from researchers. This approach is valuable for expressing hard-to-express proteins, as constitutive viral promoters are susceptible to silencing and cell instability, especially under stressful conditions (Yee et al., 2009; Lin et al., 2015; Xu et al., 2019). However, it is worth noting that culturing cells under physiological temperature downregulates the expression of specific genes, such as ribosomal protein S6 kinase (S6k) and tumor translationally-controlled 1 (Tpt1), which regulate cellular growth and proliferation, often in conjunction with the mammalian target of rapamycin complex 1 (mTORC1) (Yoon et al., 2006).

**FIGURE 1:**
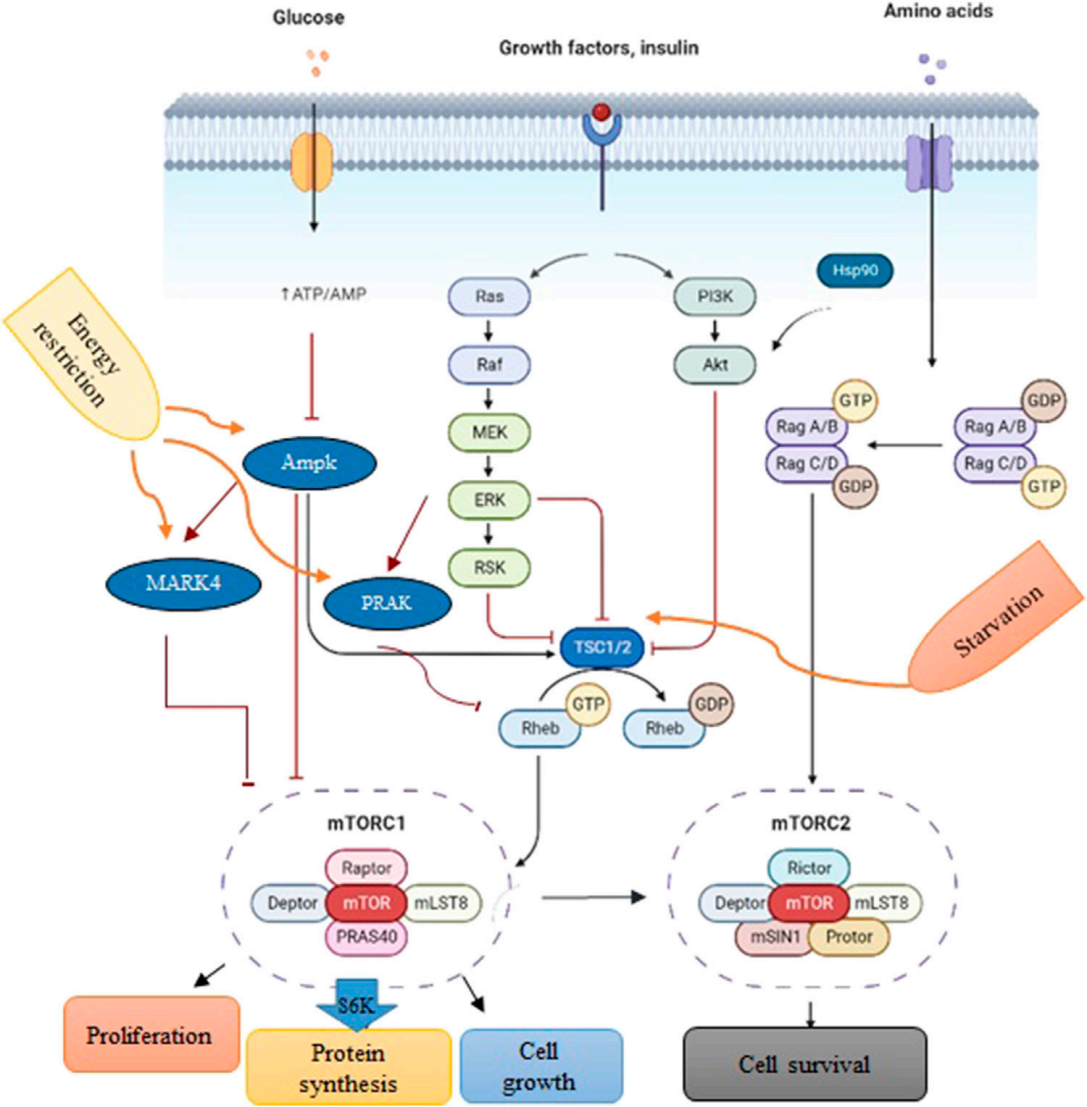
A schematic depiction of *PI3K/Akt/mTOR* signaling. Environmental conditions and growth factors regulate cell growth, proliferation, metabolism, and survival through the interaction of upstream proteins *by the mTORC1* complex. Biorender software was used to create the figure.

In cells, mTORC1 is an evolutionarily conserved serine/threonine protein kinase that regulates protein synthesis, cell growth, and proliferation in response to the surrounding environment (Figure 1) (Jossé et al., 2016; Saxton and Sabatini, 2017). The activity of mTORC1 is positively controlled by growth signals such as insulin-like growth factor (IGF). However, it can be inhibited by tuberous sclerosis 1 (*TSC1*) in response to the nutrient deficit (McVey et al., 2016; Lai et al., 2021), AMP-activated protein kinase (*AMPK*) during energy limitation or a reduction in the ATP/AMP ratio (Li et al., 2014), mitogen-activated protein kinase-activated protein kinase 5 (*MAPKAPK5*) upon energy starvation (Cully et al., 2010; Zheng et al., 2011; Ronkina et al., 2015; Han et al., 2020), and MAP/microtubule affinity-regulating kinase 4 (*MARK4*) due to a drop in energy levels (Li and Guan, 2013).

Several investigations have indicated a direct correlation between mTOR activity and CHO productivity (Edros et al., 2014). However, its role in responding to cold shock in CHO cells as producers of recombinant proteins has yet to be examined. Thus, our research aims to determine whether TS influences the expression of mTORC1 signaling genes. Subsequently, we evaluated if the downregulation of these stress-response genes, either constitutively or in response to cold, can change mTORC1 signaling genes, cell proliferation, cell size, cell cycle, and the production of GM-CSF in stable CHO cells following TS.

## 2 Materials and methods

### 2.1 Plasmids expressing TSC1, AMPK, PRAK, and MARK 4shRNAs under control of HSP90 and CMV promoters

**FIGURE 2:**
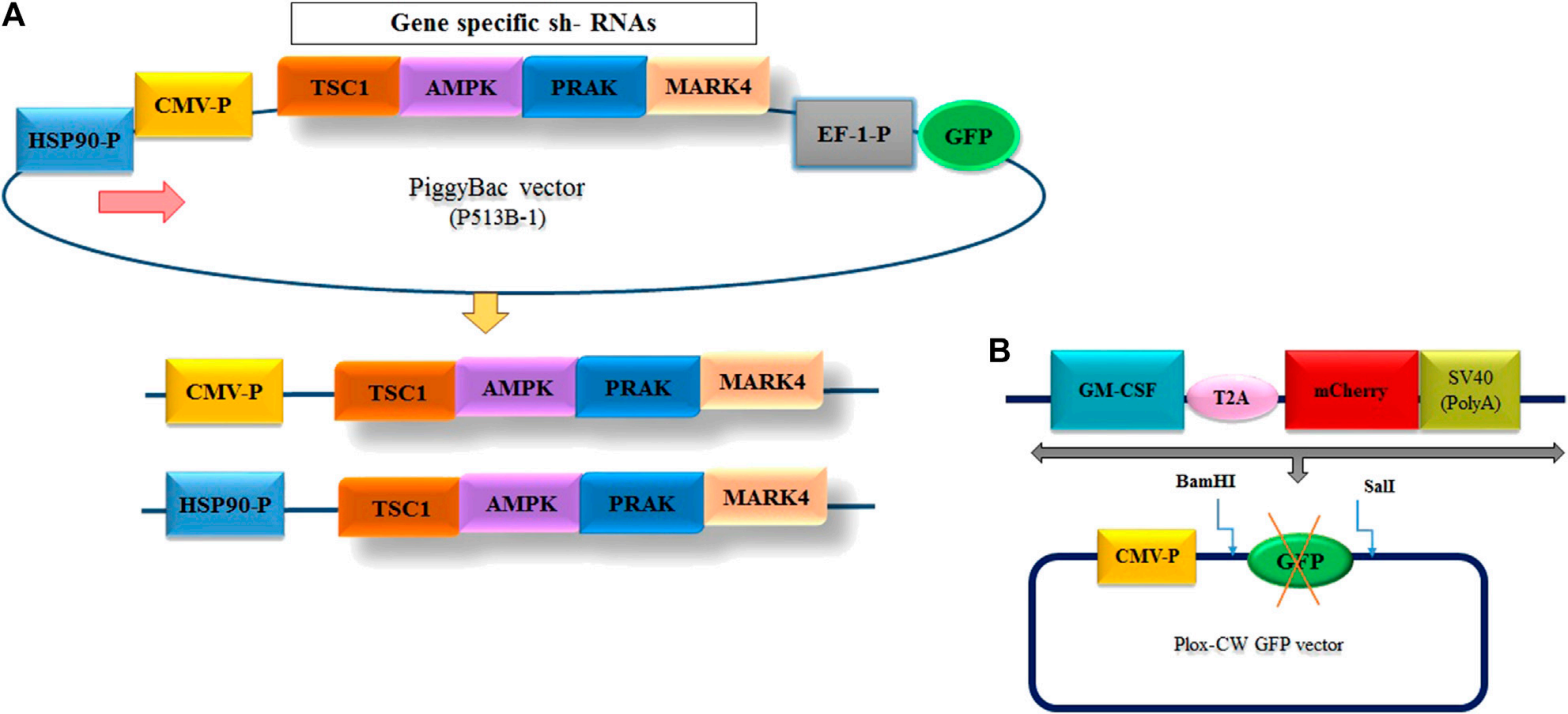
**(A)** Design of the entry vector and cloning of the multiple shRNA cassette. The diagram illustrates the structure of the entry vectors employed for shRNA cloning. To minimize the background of the parent vector during shRNA cloning, two different promoter options, CMV and HSP90—, were utilized to generate the vectors. **(B)** Schematic representation of the inserted fragment into PLOX-CW GFP.

To reduce the expression of *mTORC1* inhibitory genes at two different temperatures, we designed multiplexed shRNA targeting a combination of “*TSC1^1^/AMPK^2^/PRAK^3^/MARK^4^*” genes. The sequences of these genes, along with the HSP90 promoter, were obtained from NCBI (Supplementary Table S2). The shRNAs targeting these genes were designed using InvivoGen’s siRNA Wizard™ tool. The designed sequence was finally synthesized by GeneScript Company (China). Subsequently, these shRNAs were cloned into the piggyBAC plasmid, PB513B-1, with expression driven by either the constitutive CMV promoter or the cold-inducible HSP90 promoter (Nguyen et al., 2020) (Figure 2A). We constructed three separate plasmids, as illustrated in Figure 2A, using digestion and ligation techniques. The first plasmid, “pB513-b CMV-P/4shRNAs/GFP,” expressed a multiple shRNA cassette driven by the CMV promoter. The second plasmid, “pB513-bHSP90-P/4sh-RNAs/GFP,” contained a multiple shRNA cassette driven by the HSP90 promoter (Figure 2A). The last plasmid served as a backbone as mock group, where all pB513B-1 and shRNA sequences were removed. The ScreenFect^TM^A reagent (FUJIFILM, Japan) was used to transfect these three plasmids into GM-CSF-producing CHO-K1 cells.

### 2.2 Cell culture

The CHO-K1 and HEK293T cell lines were obtained from the Pasteur Institute (Tehran, Iran). All cell lines were cultured in DMEM-F12 and DMEM media (GIBCO, Life Technologies Inc., United States) supplemented with 1% (v/v) penicillin/streptomycin and 10% (v/v) heat-inactivated fetal bovine serum at 37°C in a humid atmosphere with 5% CO_2_. The cells were cultivated at 37°C until they reached the exponential growth phase for the TS studies. They were then incubated in a humid environment with 5% CO_2_ in the air at 30°C.

### 2.3 Generation of stable CHO cell line expressing GM-CSF

To generate the CHO cell line with constant expression of *GM-*CSF, the GM-CSF-T2A-mCherry fragments were amplified by PCR from the “PUC57, CMV-P-GM-CSF-mCherry-SV40 poly A signal” plasmid (Thesis by ethical code: IR. GOUMS.REC.1399.092), which was available in our laboratory (Figure 2B) using primers listed in Supplementary Table S1. Following amplification, the fragments were purified, digested with *BamHI* and *SalI* restriction enzymes (Vivantis, United States) and subcloned into the “pLOX-CW GFP” transfer plasmid at the same restriction enzyme sites. A second-generation lentiviral packaging system was used for constructing viruses carrying the *GM-CSF* sequence. To produce viral particles, HEK293T cells were co-transfected in 60 mm Petri dish using ScreenFect ™ as manufacturer instructions with the lentivirus transfer vector (Plox-WC-GMCS-F-mCherry), psPAX2 and pMD2G (Thermo Fisher Scientific, United States) at 3:2:1 ratio respectively. Next day, Transfection was confirmed via fluorescence microscopy to observe the expression of the *mCherry* dye in transfected cells. After 48 and 72 h of transfection, cell culture supernatants were collected, pooled, filtered through a 0.45 mm pore size filter to remove cell debris, and then stored at −80°C. Twenty-4 h before transduction, 6 × 10^4^ CHO-K1 cells were plated in 24-well culture plates. Following 48–72 h of transduction, fluorescence microscopy was used to observe *mCherry* expression (Supplementary Figure S1). Finally, to select CHO-K1 cells with the GM-CSF transgene, transduced cells were serially diluted in 96-well plates (Supplementary Figure S2). After 2 weeks, single-cell clones were isolated, identified and subcultured into 24-well plates. Afterwards, the expression of GM-CSF was measured using ELISA kit.

### 2.4 mRNA quantification by RT-qPCR technique

The total RNA was extracted from transfected cells using a Trizol reagent (Bio Basic, Canada) according to the manufacturer’s protocol. cDNA synthesis was carried out using the Yekta Tajhiz Azma cDNA Synthesis Kit (YT4500; Tehran, Iran). The real-time RT-qPCR analysis was performed using the LineGeneK Fluorescence qPCR machine (Hangzhou Bioer Technology, China), with β-actin as the internal control. Each experiment was conducted in triplicate. The primers utilized in RT-qPCR are listed in Supplementary Table S2.

### 2.5 Total protein assessment

The QuantiPro BCA Assay Kit (Sigma-Aldrich, Ireland) determined the total protein content. 4 × 10^5^ stable cells were cultured in 6-well plates and transfected with three different constructs. The transfected cells were maintained at two different temperatures: 30°C and 37°C. Subsequently, the cells were detached and centrifuged at 500 g for 5 min. A protease inhibitor cocktail and cell lysis reagent (RIPA) buffer were utilized to disrupt the cells. The samples were vortexed for 1 min and then centrifuged at 15,000 g for 15 min at 4°C. The supernatant was transferred into a new microcentrifuge tube and stored at −20°C.

### 2.6 mTOR protein detection assay

Western blot analysis was carried out to detect mTOR and β-actin proteins on 6% and 12% SDS–PAGE gels, respectively. Briefly, proteins were electrotransferred onto a nitrocellulose paper. After blocking for 1 h in TBS with 0.1% Tween 20 (TBST) and 5% skim-milk, the membranes were incubated overnight with mouse anti-mTOR primary antibody (proteintech) and mouse anti-β-actin primary antibody (Santa Cruz Biotechnology, INC) in TBST containing 1% skim milk. Subsequently, the secondary antibody, HRP-conjugated anti-mouse IgG, was applied (BioLegend). Finally, the detection was performed using an enhanced chemiluminescence (ECL) kit (Santa Cruz Biotechnolog).

### 2.7 Enzyme-linked immunosorbent assay for determination of GM-CSF production

The amount of secreted GM-CSF in the culture supernatant was measured using the human GM-CSF ELISA (MAX TM Deluxe Set) kit, employing a sandwich ELISA technique. Stable CHO cells (9 × 10^4^cells per well of a 12-well plate) were transiently transfected with shRNAs targeting *mTORC1* inhibitor genes and control plasmids. This allowed us to determine the effects of transient knockdown of these genes on GM-CSF concentration at both low and standard temperatures. After 72 h, the cell culture supernatant was harvested from all tested groups, with the concentration of secreted GM-CSF evaluated using the GM-CSF ELISA kit.

### 2.8 Cell proliferation assay

To measure the proliferation rate of the different experimental groups, 10,000 CHO- GM-CSF cells were seeded in 24-well cell culture plates. Following the transfection of stable CHO cells with the three constructs described above, cell counting was performed using a hemocytometer counter every 24 h for 3 days at both temperatures. The proliferation profiles are the average readings from at least three independent experiments, with error bars representing the standard deviation.

### 2.9 Cell cycle assay

Changes in cell-cycle distribution were determined after temperature downshift and transfection with target plasmids. CHO-GM-CSF cells were stained with propidium iodide (San Diego, California, United States) to evaluate DNA content. Cells were harvested 72 h after transfection, rinsed with cold PBS, and fixed with 70% ethanol for 2 h at −20°C. The fixed cells were then suspended in the PBS containing 100 μg/mL RNase A and incubated at 37°C for 30 min to remove intracellular RNA. Following this, the cells were washed to remove the RNaseA solution. Next, the cells were stained with propidium iodide (PI) (50 μg/mL) in a dark environment for 15 min. The proportions of cells in the G0/G1, S, and G2/M phases in the different experimental groups were evaluated using a BD FACSCalibur flow cytometer (BD Biosciences, United States). Finally, the acquired data were analyzed using ModFit™ LT6 (Bedford, United States).

### 2.10 Cell size determination

3 × 10^5^ CHO- GM-CSF cells were seeded into 10 cm^2^ cell-culture dishes and transfected with three constructs to measure the cell size. The cells were then cultivated for 72 h at 30°C and 37°C. Prior to detachment, the cells were washed with 5 mL of PBS and incubated for 5 min at room temperature in 1 mL of PBS-EDTA. The collected cells were washed twice with 10 mL of ice-cold PBS for forward light scatter (FSC) analysis, correlating with the cell size. The cell size was assessed using flow cytometry in all experimental groups. The flow cytometry profiles were analyzed using FlowJo software (Tree Stat, Inc., Ashland, United States).

### 2.11 Measuring cell death by propidium iodide

The range of cell death in the different experimental groups was determined using propidium iodide staining, following the manufacturer’s protocol. Briefly, 72 h after transfection of the constructs and culturing the cells at two different temperatures, the cells were harvested by trypsinization, suspended in PBS, and then stained with propidium iodide for 15 min in the dark at room temperature. This staining approach enabled the detection of late-stage apoptosis and necrosis. The stained cells were immediately analyzed using a BD FACSCalibur flow cytometer, with the acquired data further analyzed using FlowJo software.

### 2.12 Statistical analysis

The statistical analysis was conducted using GraphPad Prism 6 software (version 8.1). Mean differences were analyzed using the Student’s *t*-test. A significant level of less than 0.05 was used as statistical significance (*p* < 0.05).

## 3 Results

### 3.1 TSC1/AMPK/PRAK/MARK4 mRNA levels increased upon temperature shift

**FIGURE 3:**
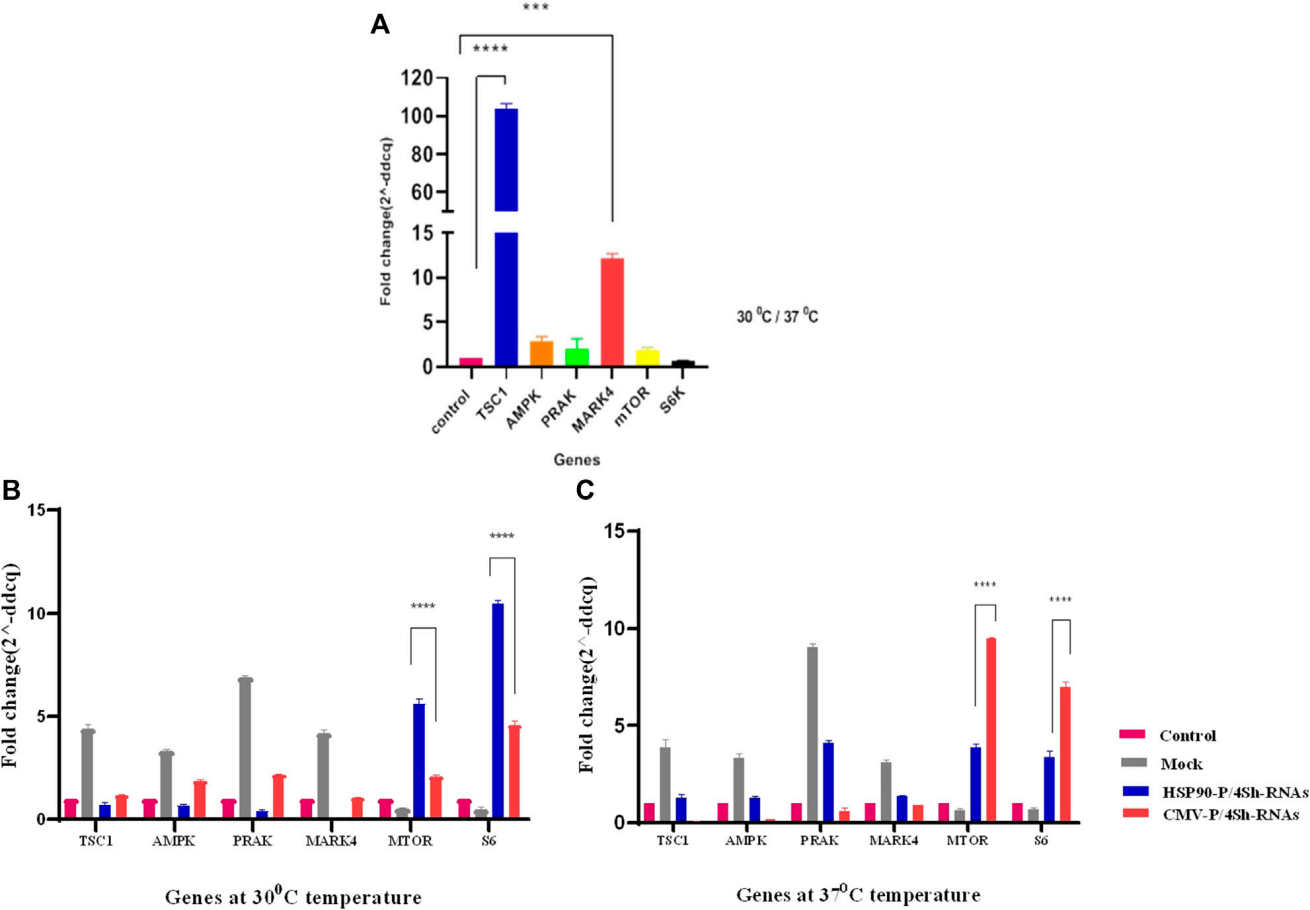
**(A)** Comparison of gene expression under lowered cultivation temperature versus standard temperature. **(B,C)** Gene expression analysis of stable CHO-K1 cells using shRNA expression vectors. The silencing efficacy of HSP90 and CMV promoters was compared at 37°C temperature. Additionally, the silencing efficacy of HSP90 promoter-induced shRNA, and CMV promoter-induced shRNA was assessed conditionally at 30°C temperature. Control is untransfected cells. Mock is cells transfected with the backbone plasmid. The data for expressed genes are presented as relative values (means ± SD).

The effect of TS on the expression of mTORC1 signaling pathway was evaluated in GM-CSF recombinant CHO-K1 cells using real-time PCR. As shown in Figure 3A, in response to TS, the expression of negative regulators of mTORC1, especially TSC1, increased by more than 100-fold (*p*-value > 0.001 significant level) the comparison with the control group (CHO cell in normal condition). Additionally, there was a 50% reduction in the S6k transcript level, while no significant changes were observed in the expression of the mTOR gene (Figure 3A).

### 3.2 Effect of lowering the temperature on HSP90 and CMV promoter activity

The stable CHO-K1 cells were transfected with HSP90-4shRNA, CMV-4shRNA, and the backbone plasmids as a mock group, allowing for the evaluation of effects caused by transfection reagents such as expression of negative control mTORC1 genes and background auto-fluorescence noise at two different temperatures to assess the knockdown efficiency of the plasmids. As shown in Supplementary Figure S3, the transfection efficiency of the constructs ranged within 50%–60% of the cell. RT-qPCR analysis revealed that both constructs significantly reduced the expression of target genes at both temperatures. However, as indicated in Supplementary Table S3, the gene expression analyses revealed that shRNA driven by the CMV promoter primarily resulted in a 64% reduction in the expression of target genes, while the HSP90 promoter downregulated these genes by 89%. Moreover, it was observed that at the lower temperature, the HSP90 promoter activity was generally higher than the CMV promoter activity, while the CMV promoter appeared to be more effective at 37°C. Consequently, the expression of mTOR and S6k genes increased by 5–10 times with the HSP90-4shRNA plasmid at 30°C and by7–9 times with the CMV-4shRNA plasmid at 37°C (Figures 3B, C).

### 3.3 Activating the mTORC1complex by knocking down TSC1, AMPK, PRAK and MARK4 genes

**FIGURE 4:**
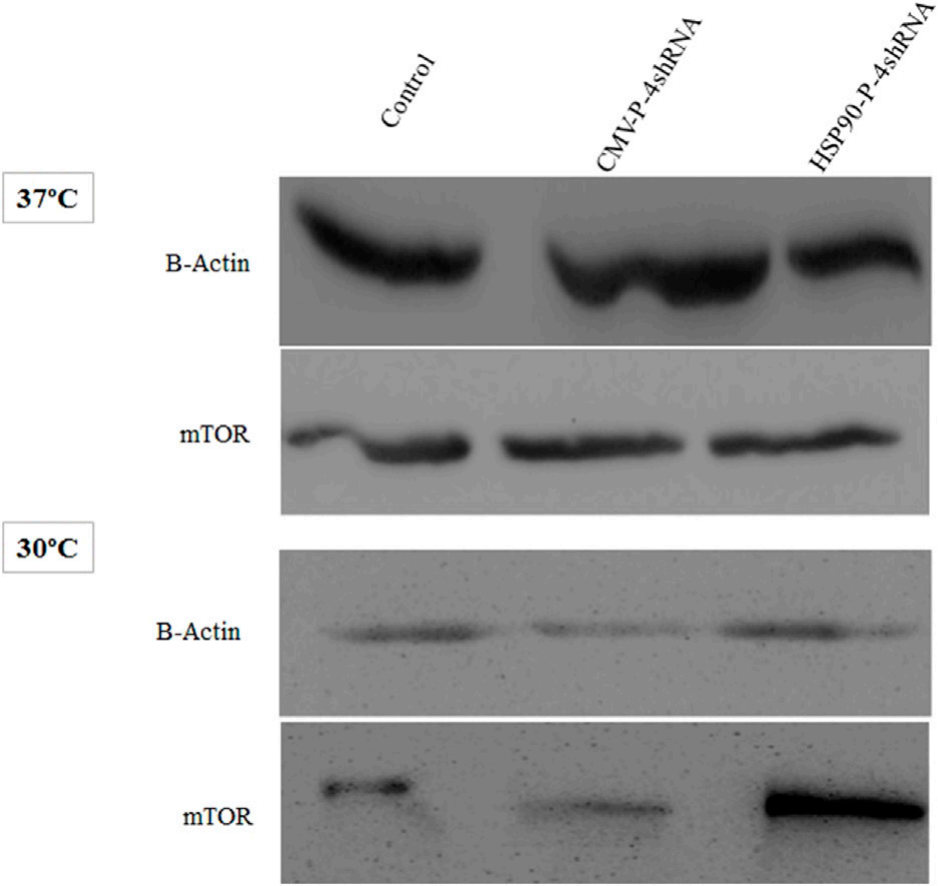
Immunoblot detection of mTOR in recombinant CHO-K1 cells expressing GM-CSF. Cell lysates were prepared from cells transiently transfected with HSP90-P-4shRNA and CMV-P-4shRNA plasmids at 37°C/30°C. The HSP90-P-4shRNA plasmid induced higher expression of mTOR protein at low temperature.

As noted above, the mTORC1 is a component of the PI3K pathway that is an upstream target of S6K and plays an essential role in protein synthesis. Here, western blot analysis demonstrating silencing effect of (TSC1, AMPK, PRAK, MARK4) genes on the total protein level of mTORC1, including both its active and inactive forms, under conditions of moderate hypothermia and standard temperature via two promoters. In comparison to the CMV-P-4shRNA-treatment, cells treated with HSP90-P-4shRNA demonstrated significantly elevated levels of mTOR protein. Conversely, in the control group exposed to low temperatures, mTOR protein levels were notably reduced (Figure 4).

### 3.4 Reducing expression of mTORC1 negative regulators enhances protein synthesis and GM-CSF production

**FIGURE 5:**
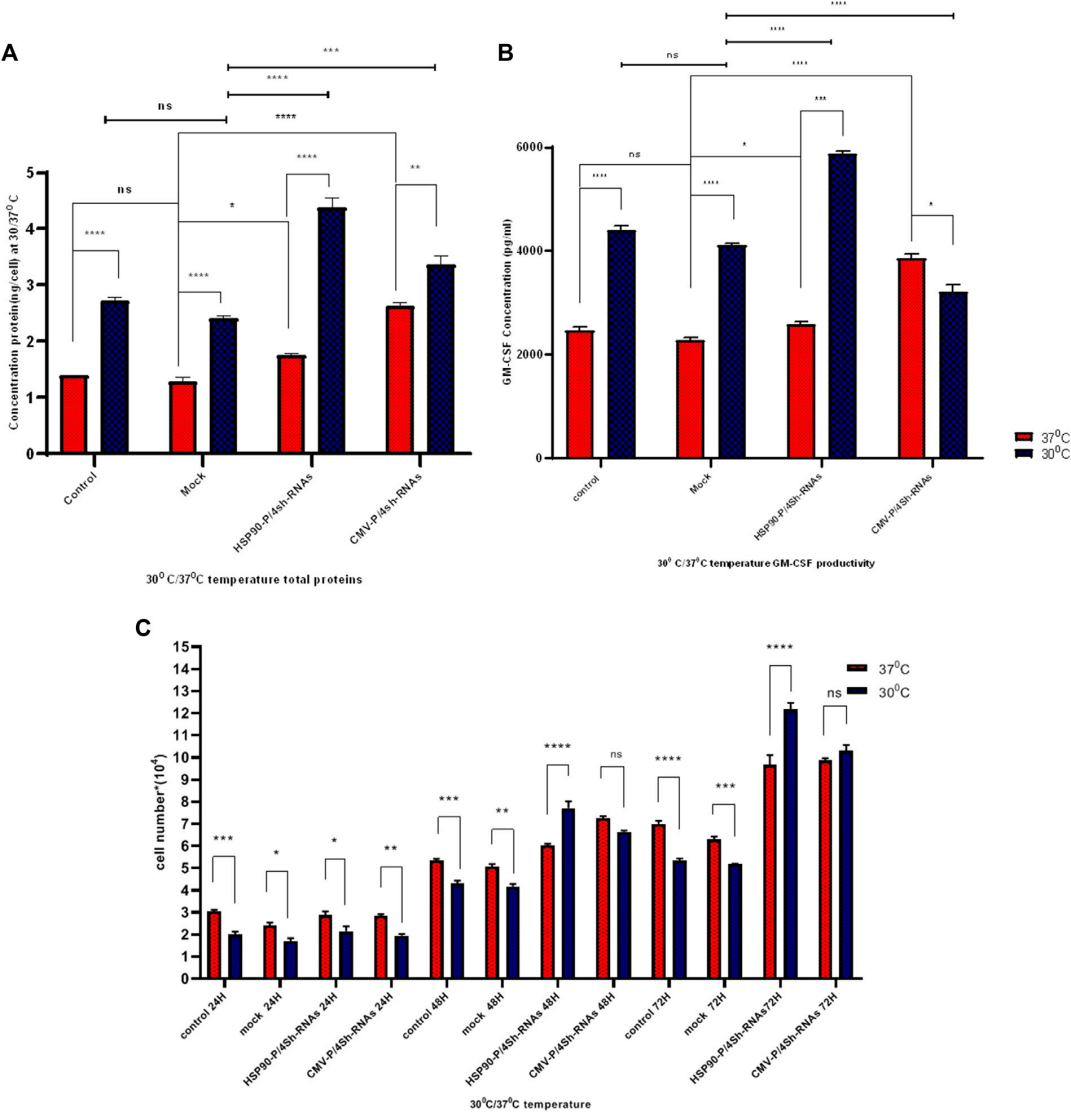
**(A)** Protein determination under different promoters and temperature conditions. The influence of temperature shift from 37°C to 30°C on stable GM-CSF recombinant CHO-K1 cells transfected with shRNAs against TSC1, AMPK, PRAK, and MARK4 was examined. Cells were harvested at indicated time points (h) after the temperature shift. **(B)** ELISA detected GM-CSF concentration at standard and low temperatures. GM-CSF concentration was measured after transfection with untreated, mock, HSP90-P/4sh-RNA, and CMV-P/4sh-RNA in 72 h, comparing standard (37°C) and low (30°C) temperatures. **(C)** Impact of mTORC1 inhibitor genes downregulation on cell proliferation at standard and low temperatures. The graphs represent cell proliferation in stable CHO-K1 cells at 24, 48, and 72 h after transfection. The standard temperature is 37°C (red lane), and the low temperature is 30°C (blue lane). The error bars represent mean ± SD. Statistical significance is *p-value < 0.05, ***p-value < 0.001, using a two-tailed *t*-test.

The impact of transient knockdown of mTORC1 negative regulator genes on global and specific protein production, specifically GM-CSF protein, was assessed by transfecting shRNA expressing and control plasmids into CHO-GM-CSF cells at two different temperatures. The total protein content of cells transfected with HSP90-P-4shRNA and CMV-P-4shRNA plasmids at 37°C was 1.7 ng/cell and 2.5 ng/cell, respectively, while TS increased these values to 4.2 ng/cell and 3.2 ng/cell (Figure 5A). Furthermore, our data showed that manipulating the mTORC1 pathway using a cold-inducible promoter at 30°C resulted in at least a 3-fold increase over the mock group at 37°C (*p*-value < 0.001) in GM-CSF production. Meanwhile, engineering with a constitutive promoter positively affected GM-CSF protein production, mainly at 37°C (Figure 5B).

### 3.5 Proliferation of CHO- GM-CSF cells enhanced through downregulation of mTORC1 negative regulator genes

The proliferation of CHO-GM-CSF cells was investigated upon cell transfection with three plasmids at low and standard temperatures using a counting assay at 3 days (Figure 5C). The findings indicated that temperature alone lowered the proliferation of CHO- GM-CSF cells. However, the downregulation of mTORC1 negative regulator genes by either plasmid after 2 days significantly elevated the proliferation rates of cells in both temperatures. This effect was particularly pronounced at 30°C when cold-induced promoters were utilized for cell engineering (Figure 5C).

### 3.6 Impact of mTORC1 signaling manipulation on cell size

**FIGURE 6:**
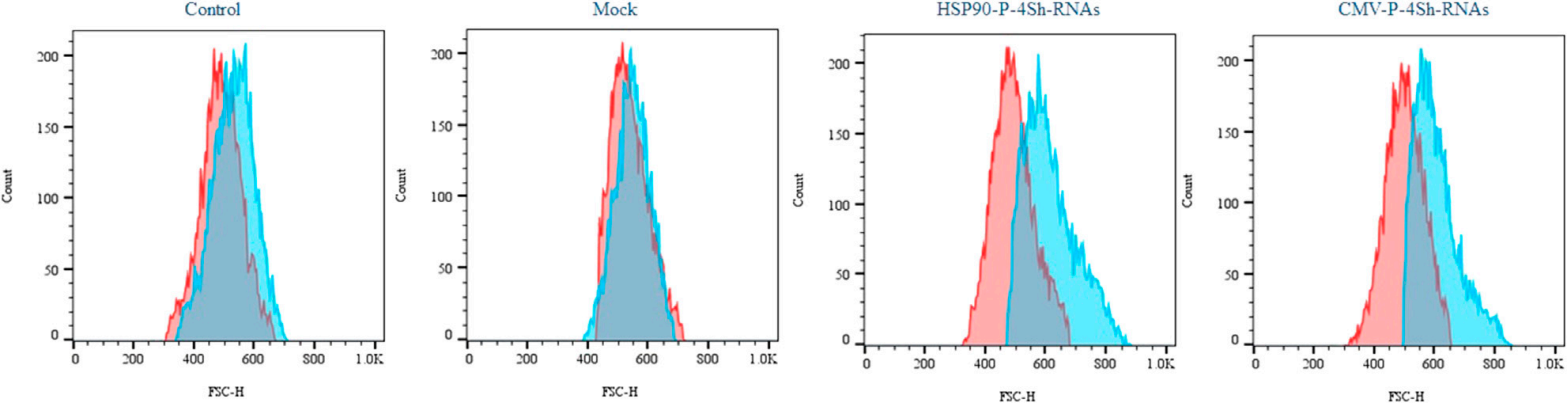
Impact of activation of mTORC1 on cell morphology, Semi-quantitative cell size profiles resulting from analysis of the FSc of at least 200,000 transfected cells in standard temperature and low temperature (red graph 37°C and blue graph 30°C).

We conducted a cell size analysis using flow cytometry across different groups to evaluate the impact of mTORC1 signaling modulation and low temperature on the growth of stable CHO-k1 cells. As depicted in Figure 6, the reduction of the temperature alone did not significantly increase the cell size. However, when TS was combined with the modulation of mTORC1 regulator genes, a notable increase in cell size was observed.

### 3.7 Modulation of mTORC1 signaling at 30°C induces cell cycle progression from G1 to G2/M phase

**FIGURE 7:**
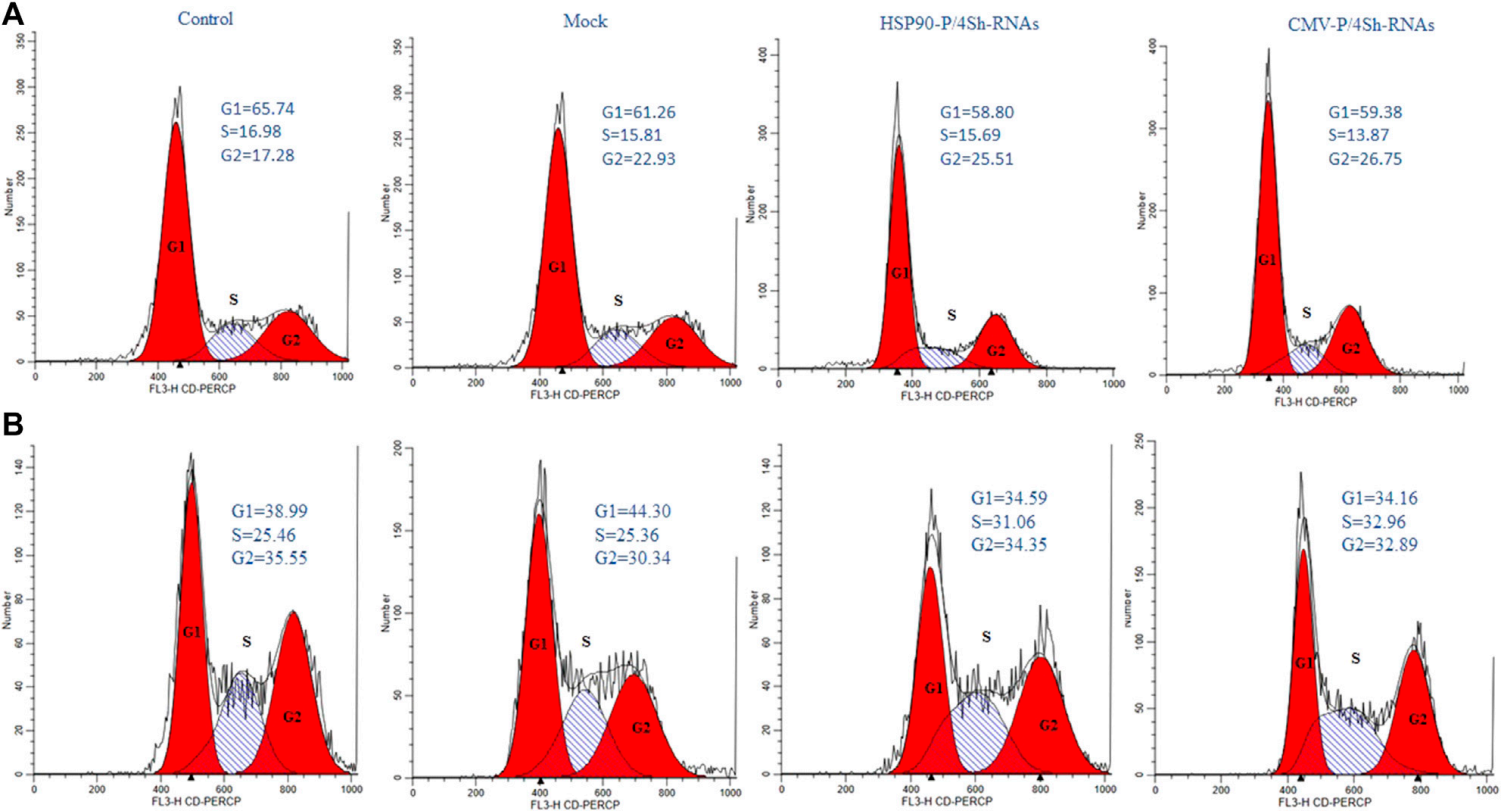
Cell-cycle distribution of recombinant CHO-K1 cells. **(A)** 72 h after temperature shift, **(B)** 72 h standard temperature, stained with propidium iodide and analyzed for their DNA content by flow cytometry.

To further explore the effect of TS and mTORC1 modulation on the cell cycle distribution in GM-CSF expressing cells, flow cytometry analysis was performed 72 h after transfections. All groups were stained with PI and evaluated using FlowJo software. The cell cycle analysis showed that in cells cultured at low temperatures, 65% of cells were located in the G1 phase, while 16% remained in the S phase and 17% in the G2/M phase (Figure 7A). However, upon transfection with inhibitory plasmids compared to the backbone plasmid (mock group), both inhibitory plasmids caused an increase in cells in the G2/M phase (Figure 7A). A slight shift to the S phase was also observed at 37°C upon modulation of mTORC1 signaling (Figure 7B).

### 3.8 Assessing cellular viability

**FIGURE 8:**
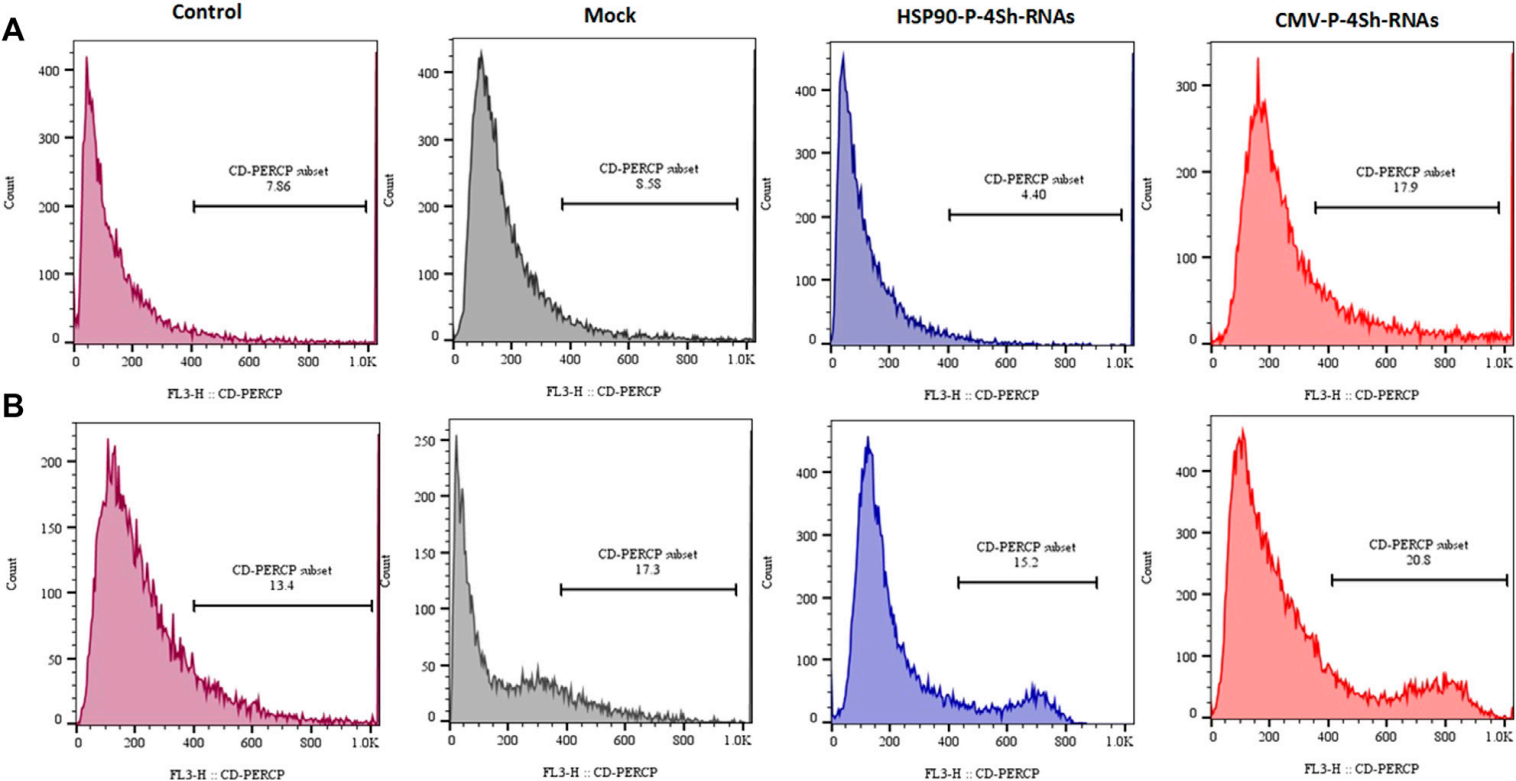
Mortality percentage, **(A)** Mortality percentage and 72 h (temperature shift) **(B)** 72 h at 37°C temperature finally stained with propidium iodide and analyzed for their DNA content by flow cytometry.

The analysis of cell viability in our study involved assessing DNA content through flow cytometry and subsequent analysis using FlowJo software. We observed a significant reduction in cell mortality, with an approximate 14% decrease upon lowering the temperature. Additionally, at the 72-h time point, downregulating mTORC1 negative regulator genes through the HSP90 promoter in TS cells resulted in a minor decline of less than 4.5% in the cell death rate (Figure 8A). Conversely, manipulating mTORC1 signaling via the CMV promoter led to an increased death rate of 18% at 30°C. Interestingly CMV-4shRNA vector showed high rate of mortality especially at 37C in the cells (Figure 8B).

## 4 Discussion

The present study aimed to investigate the impact of TS (temperature shift) and modulation of mTORC1 signaling on various cellular processes in GM-CSF-expressing cells. Our primary objective was to enhance the productivity of CHO-GM-CSF cells by focusing on mTORC1 signaling, which plays a crucial role in regulating cell growth in response to external stimuli. The results of this study provide significant insights into cell cycle distribution, cell viability, and cell size in response to temperature variations and manipulation of mTORC1 negative regulator genes.

To gain further insights into the underlying mechanisms of these changes in the cell growth behavior induced by temperature stress, we specifically investigated the effect of cold shock, an environmental factor known to improve protein properties and boost protein production (Moore et al., 1997; Roobol et al., 2020). Consistent with previous studies, we observed that mild cold exposure resulted in an overall increase in the total protein content, GM-CSF production, and cell viability. Additionally, the cold shock condition led to a higher number of cells exiting the proliferation cycle and entering the G1 phase, indicating altered cell cycle dynamics. Furthermore, to elucidate the molecular basis of these temperature-induced changes in cell growth, we examined the expression of mTORC1 signaling-related genes, including TSC1, AMPK, PRAK, and MARK4, as well as the mTOR and S6k genes, in GM-CSF producing CHO cells. Our findings indicated that culturing cells under physiological conditions resulted in differential upregulation of mTORC1 negative regulator genes and a 50% reduction in S6k levels. However, we did not observe any significant changes in the level of the mTOR transcript. Nevertheless, previous studies have demonstrated that modifying the expression of stress-responding genes, mainly by influencing S6k and mTORC1 activity, can effectively regulate cell behaviors (Tee et al., 2002; Zheng et al., 2011; Li and Guan, 2013; Liu et al., 2016). The lack of significant changes in the mTORC1 transcript levels despite the observed upregulation of mTORC1 negative regulator genes and a reduction in S6k levels can be attributed to several factors. Firstly, post-transcriptional regulation plays a significant role in controlling mTORC1 activity. It includes phosphorylation and protein degradation, which can modulate mTORC1 function without altering mRNA levels. Additionally, the mTOR pathway is a highly complex and tightly regulated network where compensatory mechanisms may come into play. It is not unusual for changes in one part of the pathway to be balanced by alterations in other components. While intriguing, the lack of significant mTORC1 transcript level changes is consistent with the intricate regulatory mechanisms governing this pathway.

Based on these findings, we can conclude that cold shock, which induces the expression of responsive genes while negatively affecting S6k and mTORC1 activity, suppresses cell growth. Thus, in our study, we aimed to improve cell proliferation and protein synthesis rates in stable CHO-K1 cells cultured at low temperatures by lowering the expression of TSC1, AMPK, PRAK, and MARK4, thereby maintaining an active mTORC1 complex. As the choice of a promoter to drive the expression of target genes is a critical step in cell line engineering, the HSP90 and CMV promoters were selected for genes as mentioned above As, targeting the genes as mentioned (Nguyen et al., 2019; 2020). The CMV promoter is a strong viral promoter commonly used in mammalian expression vectors for constitutive gene expression (Nguyen et al., 2019). However, it has potential drawbacks, such as gene silencing, apoptosis, and clonal instability, especially under cellular stress conditions (Thaisuchat et al., 2011; Misaghi et al., 2014; Romanova and Noll, 2018). On the other hand, the HSP90 promoter is an endogenous promoter that primarily drives gene expression in response to cold shock (Yang and Paschen, 2008; Nguyen et al., 2019). Following the transfection of the stable cells with these two constructs, we found that shRNAs driven by the HSP90 promoter achieved at least 30% more effective silencing of the target genes than those driven by the CMV promoter at low temperatures. However, the CMV exhibited superior performance to the HSP90 promoter at 37°C (Figures 3B, C). Interestingly, although the CMV promoter was 30% weaker than the HSP90 promoter at low temperatures, it resulted in a 14% higher cytotoxicity in CHO cells. However, it did not significantly alter the cell viability compared to the control groups at 37°C. Notably, a study also highlighted the superiority of the HSP90 promoter over the CMV promoter in CHO cells at low temperatures, 33°C, demonstrating its stability during long-term culture (Nguyen et al., 2019; Nguyen et al., 2020).

Based on these findings, we propose the HSP90 promoter as a safe and effective choice for inducing gene expression in low-temperature and bioreactor environments. Its ability to enhance gene silencing and stability makes it a promising option for optimizing protein production in stable CHO cells. Further investigations are warranted to elucidate the underlying molecular mechanisms and to optimize the use of HSP90 promoter in biotechnological applications. Our gene manipulation experiments demonstrated that reducing stress-responding genes resulted in the activation of both S6k and mTOR genes and increased mTOR protein levels, regardless of the temperature conditions. This finding aligns with prior studies highlighting the regulatory role of stress-responding genes in controlling the activity of S6k and mTORC1 (Wu and Storey, 2021; Latorre et al., 2023). Stress-responding genes influence not only the activity but also the expression of S6k and mTORC1. Interestingly, our results suggest that transient reduction of mTORC1 negative regulator genes in GM-CSF-producing CHO cells leads to improvements in various cell features related to heterologous protein production, including cell size, proliferation rate, viability, protein content, and specific productivity, at both low and moderate temperatures. Meanwhile, the effects were particularly pronounced at low temperatures, mainly when the engineering was performed using an HSP90 promoter. This resulted in a remarkable threefold increase in GM-CSF productivity in the stable CHO cells. These findings align with earlier studies highlighting the potential of modulating mTORC1 signaling to enhance protein production in CHO cells. For instance, (McVey et al., 2016), reported a two-fold increase in specific productivity in TSC2-knockout CHO cells with hyperactive mTORC1 under fed-batch conditions. Another group of researchers achieved up to four-fold improvement in the protein yield in the bioreactor and enhanced resistance to suboptimal growth conditions by ectopically expressing human mTOR at 37°C (Dreesen and Fussenegger, 2011). Additionally, using the Rheb gene, a small GTPase that activates mTORC1 upon binding to GTP, resulted in a three-fold enhancement in luciferase production in CHO cell lines (De Poi et al., 2021). These examples highlight the potential of engineering mTORC1 signaling as a promising strategy to enhance CHO cell productivity under various conditions (Roobol et al., 2020; Wu and Storey, 2021). Nevertheless, further research is required to precisely determine the most influential genes to target and to understand the long-term consequences of sustained activation of the mTORC1 complex on CHO cells and protein quality. These considerations will be critical in optimizing and refining the application of mTORC1 signaling engineering for improved bioprocess performance and protein production in CHO cells.

While this study primarily aims to investigate stress genes related to mTORC1 signaling pathway, it is essential to acknowledge its limitations, some of which may impact the interpretation of the results. First, it is worth noting that our examination of mTORC1 signaling was conducted on a transient basis, with plans for future stable investigations. This is an essential step since one of the limitations is the potential for excessive mTORC1 activity leading to cell death (apoptosis), which is not conducive to our goal of enhancing protein production. It is crucial to determine how much mTORC1 modulation will be suitable for large-scale protein production in the industry. Another limitation is that we selected a single clone of GM-CSF-producing cells without considering the effect of GM-CSF gene copy number and production rate on mTORC1 signaling and its modulation. Previous research has indicated that cells adapt their metabolic and signaling pathways, such as glycolysis, the tricarboxylic acid cycle, cell cycle regulation, and mTORC1, according to the type and quantity of protein they produce (Edros et al., 2014; Coulet et al., 2022). This suggests that the activation rate of mTORC1 may vary depending on the percentage of protein production for each clone. We hypothesize that mTORC1 modulation might have a more significant impact on low-producer cells than on high-producer cells, as over-activity of mTORC1 can lead to cell death, likely through autophagy inhibition (Villar et al., 2017). However, testing this hypothesis across different clones is essential to better understand the trade-off between production rates and cell survival.

Furthermore, most CHO cell lines used in bioreactors are suspension cultures that they often undergo long-term cultivation in these bioreactors. This study would benefit from validation using CHO cells stably modified to contain shRNA against negative mTORC1 regulators under the control of the HSP90 promoter. This approach would enable the assessment of the effects of fermentation and bioreactor-induced stress on both protein productivity and cell growth at lower temperatures. Lastly, constructing adaptive laboratory mammalian cells remains challenging using natural selection approaches (Jukić et al., 2016; Lee and Kim, 2020). We expected the downregulation of stress-responding genes and low levels of them to hinder the negative effect of environmental difficulties and help cells to adapt easily in suboptimal conditions.

## 5 Conclusion

In conclusion, this study provided valuable insights into the complex interplay between temperature, mTORC1 signaling, and cellular processes in GM-CSF-expressing cells. The study showed the impact of cold stress and the modulation of stress-responding genes on cell growth in CHO cells cultured at low temperatures. Our results demonstrated, for the first time, that cold stress leads to a decline in cell growth in CHO cells, possibly through the upregulation of stress-responding genes. Meanwhile, we also observed that reducing these genes using endogenous promoters can effectively modulate the negative effects of cold stress on cell growth and enhance the rate of recombinant protein production. Finally, the success of this approach indicates the importance of engineering mTORC1 signaling in stable CHO to adapt to the stressful conditions of bioreactors.

## Data Availability

The original contributions presented in the study are included in the article/Supplementary Material, further inquiries can be directed to the corresponding author.

## Footnotes

1 Tuberous sclerosis 1.

2 AMP-activated protein kinase.

3 Mitogen-Activated Protein Kinase-Activated Protein Kinase 5.

4 MAP/Microtubule Affinity-Regulating Kinase 4.

## References

[B1] Al-Fageeh M. B. Marchant R. J. Carden M. J. Smales C. M. (2006). The cold‐shock response in cultured mammalian cells: harnessing the response for the improvement of recombinant protein production. Biotechnol. Bioeng. 93, 829–835. 10.1002/bit.20789 16329142

[B2] Bryan L. Clynes M. Meleady P. (2021). The emerging role of cellular post-translational modifications in modulating growth and productivity of recombinant Chinese hamster ovary cells. Biotechnol. Adv. 49, 107757. 10.1016/j.biotechadv.2021.107757 33895332

[B3] Coulet M. Kepp O. Kroemer G. Basmaciogullari S. (2022). Metabolic profiling of CHO cells during the production of biotherapeutics. Cells 11, 1929. 10.3390/cells11121929 35741058 PMC9221972

[B4] Cully M. Genevet A. Warne P. Treins C. Liu T. Bastien J. (2010). A role for p38 stress-activated protein kinase in regulation of cell growth via TORC1. Mol. Cell. Biol. 30, 481–495. 10.1128/mcb.00688-09 19917724 PMC2798466

[B5] De Poi S. P. Xie J. Smales C. M. Proud C. G. (2021). Constitutively active Rheb mutants [T23M] and [E40K] drive increased production and secretion of recombinant protein in Chinese hamster ovary cells. Biotechnol. Bioeng. 118, 2422–2434. 10.1002/bit.27748 33694218

[B6] Dreesen I. A. Fussenegger M. (2011). Ectopic expression of human mTOR increases viability, robustness, cell size, proliferation, and antibody production of Chinese hamster ovary cells. Biotechnol. Bioeng. 108, 853–866. 10.1002/bit.22990 21404259

[B7] Edros R. McDonnell S. Al-Rubeai M. (2014). The relationship between mTOR signalling pathway and recombinant antibody productivity in CHO cell lines. BMC Biotechnol. 14, 1–10. 10.1186/1472-6750-14-15 PMC3937030 24533650

[B8] Han J. Wu J. Silke J. (2020). An overview of mammalian p38 mitogen-activated protein kinases, central regulators of cell stress and receptor signaling. F1000Research 9, 653. 10.12688/f1000research.22092.1 PMC7324945 32612808

[B9] Jossé L. Xie J. Proud C. G. Smales C. M. (2016). mTORC1 signalling and eIF4E/4E-BP1 translation initiation factor stoichiometry influence recombinant protein productivity from GS-CHOK1 cells. Biochem. J. 473, 4651–4664. 10.1042/bcj20160845 27760840 PMC5147049

[B10] Jukić S. Bubenik D. Pavlović N. Tušek A. J. Srček V. G. (2016). Adaptation of CHO cells in serum-free conditions for erythropoietin production: application of EVOP technique for process optimization. Biotechnol. Appl. Biochem. 63, 633–641. 10.1002/bab.1468 26661088

[B11] Kantardjieff A. Jacob N. M. Yee J. C. Epstein E. Kok Y.-J. Philp R. (2010). Transcriptome and proteome analysis of Chinese hamster ovary cells under low temperature and butyrate treatment. J. Biotechnol. 145, 143–159. 10.1016/j.jbiotec.2009.09.008 19770009

[B12] Lai M. Zou W. Han Z. Zhou L. Qiu Z. Chen J. (2021). Tsc1 regulates tight junction independent of mTORC1. Proc. Natl. Acad. Sci. 118, e2020891118. 10.1073/pnas.2020891118 34301883 PMC8325158

[B13] Latorre Y. Torres M. Vergara M. Berrios J. Sampayo M. M. Gödecke N. (2023). Engineering of Chinese hamster ovary cells for co-overexpressing MYC and XBP1s increased cell proliferation and recombinant EPO production. Sci. Rep. 13, 1482. 10.1038/s41598-023-28622-z 36707606 PMC9883479

[B14] Lee S. Kim P. (2020). Current status and applications of adaptive laboratory evolution in industrial microorganisms. J. Microbiol. Biotechnol. 30, 793–803. 10.4014/jmb.2003.03072 32423186 PMC9728180

[B15] Li H. Min Q. Ouyang C. Lee J. He C. Zou M.-H. (2014). AMPK activation prevents excess nutrient-induced hepatic lipid accumulation by inhibiting mTORC1 signaling and endoplasmic reticulum stress response. Biochimica Biophysica Acta (BBA)-Molecular Basis Dis. 1842, 1844–1854. 10.1016/j.bbadis.2014.07.002 PMC6408939 25016145

[B16] Li L. Guan K.-L. (2013). Microtubule-associated protein/microtubule affinity-regulating kinase 4 (MARK4) is a negative regulator of the mammalian target of rapamycin complex 1 (mTORC1). J. Biol. Chem. 288, 703–708. 10.1074/jbc.c112.396903 23184942 PMC3537069

[B17] Lin C.-Y. Huang Z. Wen W. Wu A. Wang C. Niu L. (2015). Enhancing protein expression in HEK-293 cells by lowering culture temperature. PloS one 10, e0123562. 10.1371/journal.pone.0123562 25893827 PMC4404257

[B18] Liu Z. Gan L. Chen Y. Luo D. Zhang Z. Cao W. (2016). Mark4 promotes oxidative stress and inflammation via binding to PPARγ and activating NF-κB pathway in mice adipocytes. Sci. Rep. 6, 21382–21412. 10.1038/srep21382 26888669 PMC4766853

[B19] McHugh K. P. Xu J. Aron K. L. Borys M. C. Li Z. J. (2020). Effective temperature shift strategy development and scale confirmation for simultaneous optimization of protein productivity and quality in Chinese hamster ovary cells. Biotechnol. Prog. 36, e2959. 10.1002/btpr.2959 31930722

[B20] McVey D. Aronov M. Rizzi G. Cowan A. Scott C. Megill J. (2016). CHO cells knocked out for TSC2 display an improved productivity of antibodies under fed batch conditions. Biotechnol. Bioeng. 113, 1942–1952. 10.1002/bit.25951 26888596

[B21] Misaghi S. Chang J. Snedecor B. (2014). It’s time to regulate: coping with product-induced nongenetic clonal instability in CHO cell lines via regulated protein expression. Biotechnol. Prog. 30, 1432–1440. 10.1002/btpr.1970 25104235

[B22] Moore A. Mercer J. Dutina G. Donahue C. J. Bauer K. D. Mather J. P. (1997). Effects of temperature shift on cell cycle, apoptosis and nucleotide pools in CHO cell batch cultues. Cytotechnology 23, 47–54. 10.1023/a:1007919921991 22358520 PMC3449885

[B23] Nguyen L. N. Baumann M. Dhiman H. Marx N. Schmieder V. Hussein M. (2019). Novel promoters derived from Chinese hamster ovary cells via *in silico* and *in vitro* analysis. Biotechnol. J. 14, 1900125. 10.1002/biot.201900125 31271264

[B24] Nguyen L. N. Novak N. Baumann M. Koehn J. Borth N. (2020). Bioinformatic identification of Chinese hamster ovary (CHO) cold-shock genes and biological evidence of their cold-inducible promoters. Biotechnol. J. 15, 1900359. 10.1002/biot.201900359 31785035

[B25] Romanova N. Noll T. (2018). Engineered and natural promoters and chromatin-modifying elements for recombinant protein expression in CHO cells. Biotechnol. J. 13, 1700232. 10.1002/biot.201700232 29145694

[B26] Ronkina N. Johansen C. Bohlmann L. Lafera J. Menon M. B. Tiedje C. (2015). Comparative analysis of two gene-targeting approaches challenges the tumor-suppressive role of the protein kinase MK5/PRAK. PloS one 10, e0136138. 10.1371/journal.pone.0136138 26295581 PMC4546416

[B27] Roobol A. Roobol J. Smith M. E. Carden M. J. Hershey J. W. Willis A. E. (2020). Engineered transient and stable overexpression of translation factors eIF3i and eIF3c in CHOK1 and HEK293 cells gives enhanced cell growth associated with increased c-Myc expression and increased recombinant protein synthesis. Metab. Eng. 59, 98–105. 10.1016/j.ymben.2020.02.001 32061967 PMC7118365

[B28] Saxton R. A. Sabatini D. M. (2017). mTOR signaling in growth, metabolism, and disease. Cell 168, 960–976. 10.1016/j.cell.2017.02.004 28283069 PMC5394987

[B29] Sellick C. A. Croxford A. S. Maqsood A. R. Stephens G. Westerhoff H. V. Goodacre R. (2011). Metabolite profiling of recombinant CHO cells: designing tailored feeding regimes that enhance recombinant antibody production. Biotechnol. Bioeng. 108, 3025–3031. 10.1002/bit.23269 21769861

[B30] Tee A. R. Fingar D. C. Manning B. D. Kwiatkowski D. J. Cantley L. C. Blenis J. (2002). Tuberous sclerosis complex-1 and-2 gene products function together to inhibit mammalian target of rapamycin (mTOR)-mediated downstream signaling. Proc. Natl. Acad. Sci. 99, 13571–13576. 10.1073/pnas.202476899 12271141 PMC129715

[B31] Thaisuchat H. Baumann M. Pontiller J. Hesse F. Ernst W. (2011). Identification of a novel temperature sensitive promoter in CHO cells. BMC Biotechnol. 11, 51–12. 10.1186/1472-6750-11-51 21569433 PMC3118111

[B32] Villar V. H. Nguyen T. L. Delcroix V. Terés S. Bouchecareilh M. Salin B. (2017). mTORC1 inhibition in cancer cells protects from glutaminolysis-mediated apoptosis during nutrient limitation. Nat. Commun. 8, 14124. 10.1038/ncomms14124 28112156 PMC5264013

[B33] Wu C.-W. Storey K. B. (2021). mTOR signaling in metabolic stress adaptation. Biomolecules 11, 681. 10.3390/biom11050681 34062764 PMC8147357

[B34] Xu J. Tang P. Yongky A. Drew B. Borys M. C. Liu S. (2019). Systematic development of temperature shift strategies for Chinese hamster ovary cells based on short duration cultures and kinetic modeling. MAbs 11, 191–204. 10.1080/19420862.2018.1525262 30230966 PMC6343780

[B35] Yang W. Paschen W. (2008). Conditional gene silencing in mammalian cells mediated by a stress-inducible promoter. Biochem. biophysical Res. Commun. 365, 521–527. 10.1016/j.bbrc.2007.11.011 18021742

[B36] Yao G. Aron K. Borys M. Li Z. Pendse G. Lee K. (2021). A metabolomics approach to increasing Chinese hamster ovary (CHO) cell productivity. Metabolites 11, 823. 10.3390/metabo11120823 34940581 PMC8704136

[B37] Yee J. C. Gerdtzen Z. P. Hu W. (2009). Comparative transcriptome analysis to unveil genes affecting recombinant protein productivity in mammalian cells. Biotechnol. Bioeng. 102, 246–263. 10.1002/bit.22039 18726962

[B38] Yoon S. K. Hong J. K. Choo S. H. Song J. Y. Park H. W. Lee G. M. (2006). Adaptation of Chinese hamster ovary cells to low culture temperature: cell growth and recombinant protein production. J. Biotechnol. 122, 463–472. 10.1016/j.jbiotec.2005.09.010 16253368

[B39] Zheng M. Wang Y.-H. Wu X.-N. Wu S.-Q. Lu B.-J. Dong M.-Q. (2011). Inactivation of Rheb by PRAK-mediated phosphorylation is essential for energy-depletion-induced suppression of mTORC1. Nat. Cell Biol. 13, 263–272. 10.1038/ncb2168 21336308 PMC3070924

