# Supplementary material for: Enhancing productivity of Chinese hamster ovary (CHO) cells: synergistic strategies combining low-temperature culture and mTORC1 signaling engineering
Source: Front Bioeng Biotechnol. 2023 Nov 21;11:1268048. doi: 10.3389/fbioe.2023.1268048 (PMC10699326; doi:10.3389/fbioe.2023.1268048)
Supplement: Supplementary file 1 [file DataSheet1.docx]

**Supplementary File**

Enhancing Productivity of Chinese Hamster Ovary (CHO) Cells: Synergistic Strategies Combining Low Temperature Culture and mTORC1 Signaling Engineering

Farzaneh Shahabi^1^, Shahriyar Abdoli^1, 2^, Zahra Bazi^2^, Fatemeh Shamsabadi^1, 2^, Ahad Yamchi^3^, Majid Shahbazi^2, 4^

1-Medical Cellular and Molecular Research Center, Golestan University of Medical Sciences,
Gorgan, Iran
2- Department of Medical Biotechnology ,School of advanced technologies in medicine, Golestan University of Medical Sciences,
Gorgan, Iran.

3 - Department of Plant Breeding and Biotechnology, College of Plant Production Gorgan University of Agriculture Science and Natural Recourses, Gorgan, Iran.

4 -AryaTina Gene (ATG) Biopharmaceutical Company Gorgan, Iran.

* Corresponding author:

Prof. Majid Shahbazi

Medical Cellular and Molecular Research Center, Golestan University of Medical Sciences,

Shastkola road, Falsafi complex, Gorgan, Iran. Zip code: 4934174611

Tel/Fax: (+98 17) 3243 0353, 3245 1564, (+98 912) 5140 251,

[shahbazimajid@yahoo.co.uk](mailto:shahbazimajid@yahoo.co.uk), [Shahbazim@atgbio.com](mailto:Shahbazim@atgbio.com)

ORCID ID: 0000-0002-4143-8282

Supplementary Table 1: the sequence of primers used for the cloning of gene into construct.

| 5’- BamHI CGC GGATCCTACGCGACCATGCTGCT |
| --- |
| 5’- GC GTCGAC CGCCTACTTGTACAGCTCGTC SalI |

Supplementary Table 2: the sequence and properties of primers used for RT-qPCR.

| Gene name | Forward sequence | Reverse sequence | NCBI Reference Sequence |
| --- | --- | --- | --- |
| Tsc1 | AGGCTCTCCTCCCTCAGATG | ACTTCAACTGATCTTTCATTGCTG | XM_027423070.1 |
| AMPK | CGAGAAGCAGAAGCACGAC | TCATGTTTGCCCACCTTCAC | XM_027401368.1 |
| PRAK | TGTCATTGCCCAGTGTATCC | GAATCCACGACCGTTCCAG | XM_027414087.1 |
| MARK4 | ACAGGGACCTAAAGGCAGAG | AGCCCAGTGTGAACTCATTG | XM_027430905.1 |
| mTORC1 | GATTGGTTGTGGGGCTTGC | CGAGCATCTTGCCCTAAGGT | XM_027398207.2 |
| S6K | ATATGTGGCTCCATCTGTACTTG | TCCAGTTCAGCAAAGGGTC | XM_027426359.2 |

Supplementary Table 3: Comparison of Promoter Silencing Efficiency at Different Temperatures.

| 30 ^0^C | HSP90 | CMV |
| --- | --- | --- |
| TSC1 | 84% | 72% |
| AMPK | 80% | 43% |
| PRAK | 94% | 68% |
| MARK4 | 99% | 74% |
| Total | 89% | 64% |

| 37 ^0^C | HSP90 | CMV |
| --- | --- | --- |
| TSC1 | 67% | 97% |
| AMPK | 61% | 94% |
| PRAK | 54% | 93% |
| MARK4 | 58% | 70% |
| Total | 60% | 89% |

We examined the extent to which the CMV and HSP90 promoters could effectively silence target genes at two distinct temperatures, 30°C and 37°C. The results are summarized below and the differences in silencing percentages between the HSP90 and CMV promoters at 30°C and 37°C has been calculated.

**
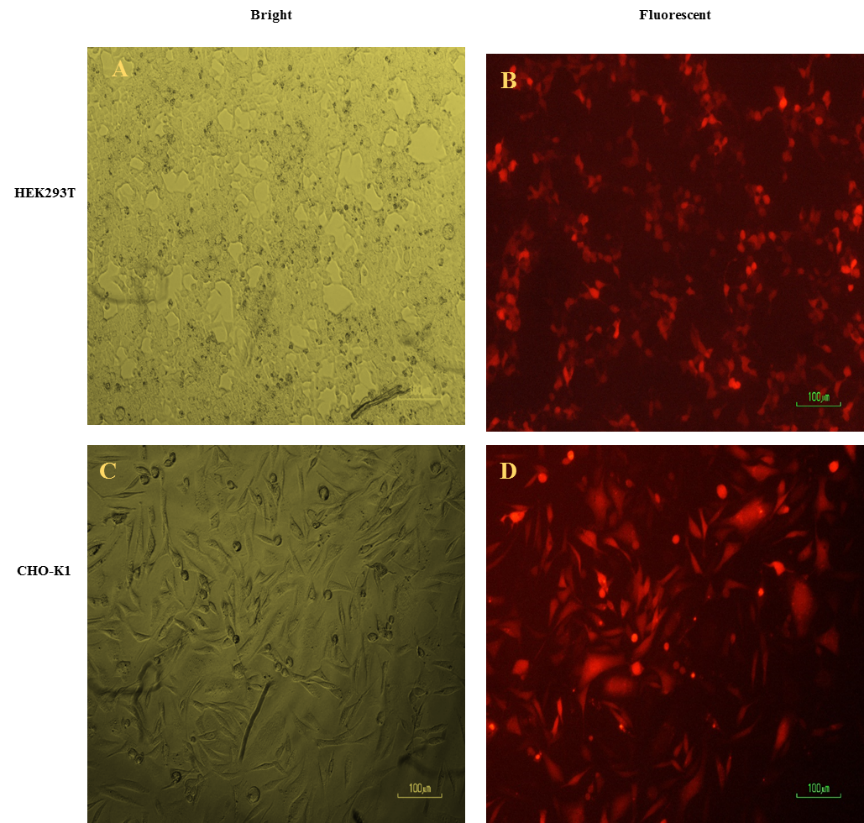
**

Supplementary Figure 1**: *(*A, B)** Fluorescent microscope image of HEK293T cells expressing *mCherry*. The lentivirus Plox-wc-CMV-P-GM-CSF-*mCherry* successfully transduced HEK293T cells **(C, D)**, and the particles of the recombinant lentivirus transduced CHO-K1 cells. CHO-K1 cells transduced with the lentivirus are shown in the image before and 72 h after transduction (Left: bright field, right: fluorescent).


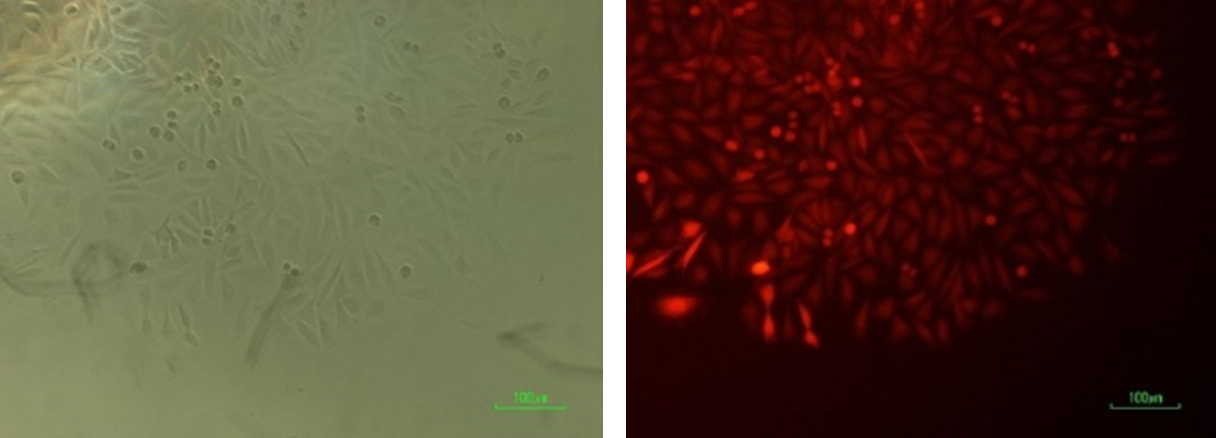


Supplementary Figure 2: single cell isolation before and after fluorescent illumination and the expression of *mCherry* was examined using the Corning procedure. Single cells were harvested 2 weeks after the transduction of GM-CSF-CHO-K1 cells.


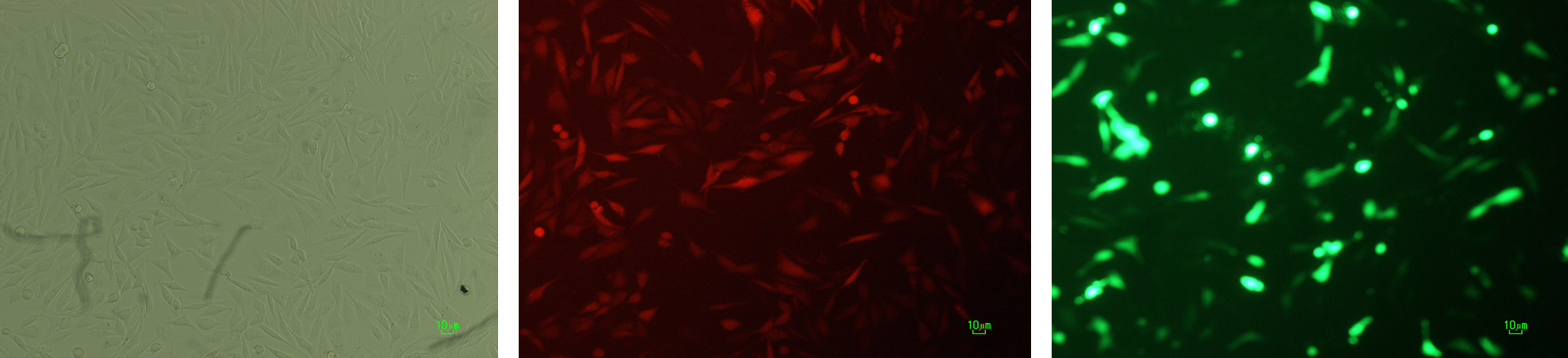


Supplementary Figure 3: The stable - CHO-GM-CSF- *mCherry* cells were transfected with HSP90-4shRNA, CMV-4shRNA, and mock plasmids including GFP as reporter gene.
